# The clinicopathological characteristics, oncologic outcomes and costs of “HER2-low” early breast cancer compared to HER2-zero and HER2-positive: a single-centre retrospective analysis

**DOI:** 10.3389/fonc.2025.1579602

**Published:** 2025-09-17

**Authors:** Patrícia Rafaela Rodrigues, Luísa Lopes-Conceição, Virgínia Sousa, Andreia Coutada, Maria José Bento, Nuno Coimbra, Conceição Leal, Deolinda Pereira, Ana Magalhães Ferreira

**Affiliations:** ^1^ Department of Medical Oncology, Portuguese Oncology Institute, Porto, Portugal; ^2^ Epidemiology, Outcomes, Economics and Management in Oncology Group - Research Center (CI-IPOP), Porto Comprehensive Cancer Center (Porto.CCC), RISE@CI-IPOP (Health Research Network), Portuguese Oncology Institute of Porto, Porto, Portugal; ^3^ Oncology Adult Day Hospital Unit, Portuguese Oncology Institute, Porto, Portugal; ^4^ Department of Anatomical Pathology, Portuguese Oncology Institute, Porto, Portugal; ^5^ Department of Population Studies, Abel Salazar Institute of Biomedical Sciences, University of Porto, Porto, Portugal; ^6^ Abel Salazar Institute of Biomedical Sciences, University of Porto, Porto, Portugal

**Keywords:** HER2-low, early breast cancer (EBC), HER2-0, HER2-positive, costs of treatment, clinic-pathlogical characteristics

## Abstract

**Background:**

Breast cancer is a heterogeneous disease commonly classified based on hormone receptor (HR) status and human epidermal growth factor receptor 2 (HER2) expression. Recently, an intermediate category termed “HER2-low” has drawn attention for its potential prognostic and therapeutic implications. This study aimed to characterize the clinicopathological features, oncologic outcomes, and costs of “HER2-low” early breast cancer (eBC) in comparison with HER2-zero (HER2-0) and HER2-positive eBC.

**Methods:**

A single-center, retrospective analysis included patients with stage I–IIIA eBC diagnosed from January 2019 to December 2020. Patients were categorized into HER2-0, “HER2-low” (IHC 1+ or IHC 2+/ISH-negative), or HER2-positive (IHC 3+ or IHC 2+/ISH-positive). Clinicopathological data, direct medical costs, disease-free survival (DFS), and overall survival (OS) were examined. Kaplan-Meier analyses compared survival outcomes among groups, and Chi-square tests assessed differences in clinical characteristics.

**Results:**

Among 1,138 patients, 35.1% were HER2-0, 45.3% were “HER2-low”, and 19.5% were HER2-positive. “HER2-low” eBC showed higher rates of HR positivity compared with HER2-0 (94% vs. 89%, p=0.018) and more frequent nodal involvement (39% vs. 30%, p=0.014). Compared with HER2-positive disease, “HER2-low” tumors presented at earlier stages, had fewer grade 3 tumors, and were less frequently treated with chemotherapy (56% vs. 83%, p<0.001). No significant differences were observed in DFS or OS among the three groups within the study’s follow-up period. Costs were highest for patients with HER2-positive eBC, primarily driven by targeted therapies (trastuzumab, pertuzumab, T-DM1).

**Conclusions:**

While “HER2-low” eBC demonstrates distinct clinicopathological features—particularly in terms of HR positivity and histological grade—this intermediate phenotype did not exhibit worse oncologic outcomes compared with HER2–0 or HER2-positive disease in the observed timeframe. Further research is needed to validate these findings and clarify the prognostic and therapeutic significance of “HER2-low” eBC, especially as new HER2-targeting agents emerge.

## Introduction

Breast cancer (BC) is the most frequent type of cancer in women globally, accounting for 32% of all cancers in this gender, and it is the second most lethal after lung cancer (1). Early BC (eBC) refers to invasive breast cancer confined to the breast with or without involvement of regional lymph nodes and no evidence of distant metastasis. Itis classified according to its histopathological factors, including expression of hormonal receptors (HR) such as estrogen (ER) and progesterone receptors (PR) and presence of human epidermal growth factor receptor 2 (HER2) overexpression, usually due to ERBB2 amplification, which is present in 15% of all BCs (2, 3). This classification divides eBC into the major subtypes: HR-positive/HER2-negative, HER2-positive and triple-negative (4).

HER2 is a member of the epidermal growth factor receptor (EGFR) family (5). Its extracellular domain has no known ligand and is activated by the formation of homo or heterodimers, which leads — via phosphorylation — to the activation of phosphatidylinositol triphosphate kinase (PI3K) and mitogen-activated protein kinase (MAPK) signaling pathways, responsible for cell cycle progression and proliferation (5).

BC is considered HER2-positive when immunohistochemistry (IHC) assay reveals HER2 overexpression (score 3+) or there is ERBB2 amplification on an *in-situ* hybridization (ISH) assay, which usually is performed if IHC shows a score of 2 +. If the IHC assay scores 0, 1+, or 2+ with a negative ISH assay, the cancer is classified as “HER2-negative” (2, 5). This classification is relevant because HER2-positive disease benefits from HER2-target therapy (5), although there is increasing evidence that an IHC score 1+ and 2+ with negative ISH may also derive clinical benefit from new agents targeting HER2, like anti-HER2 antibody-drug conjugates (ADC) (2). This is particularly notable for advanced “HER2-low” BC, in which trastuzumab-deruxtecan (T-DXD) showed anti-tumor activity in DESTINY-Breast04 trial, demonstrating that even a low to moderate HER2 expression is sufficient to obtain a therapeutic response, thereby opening a new therapeutic option (5–7).

Therefore, instead of a binary classification of HER2-negative or HER2-positive eBC, intermediate levels of expression (termed “HER2-low” eBC) could also be considered, and if so, they may represent more than half of eBC cases, since some studies report 45–60% of eBC with this phenotype. Patients with IHC score 0 are considered HER2-0 (2, 3, 5, 7, 8).

Accordingly, it is necessary to understand the prognostic significance of “HER2-low” eBC, its clinicopathological characteristics and the treatment costs associated with this disease. Thus, this study aimed to characterize the clinicopathological features, oncologic outcomes, and costs of “HER2-low” early breast cancer (eBC) in comparison with HER2–0 and HER2-positive eBC.

## Materials and methods

### Study design

We conducted a retrospective analysis of a cohort of patients diagnosed with eBC at a Portuguese Comprehensive Cancer Center between 1 January 2019 and 31 December 2020. Inclusion criteria encompassed female patients aged with 18 years-old or older at diagnosis, with histologically proven primary invasive localized BC (stages I to IIIA) and who performed all treatments at our institution. Patients were excluded if they had missing diagnostic data or missing information regarding HER2 status, were lost to follow-up, participated in clinical trials or had other malignancies (except non-melanoma skin cancer) within five years before or after the diagnosis. Therefore, a total of 1138 patients were included in the present study.

### Data collection

Data collection included patient clinical and pathological characteristics, such as age at diagnosis, menopausal status, histologic type and grade (based on core biopsy), HR status (considered positive when either ER or PR was ≥1%; negative when both ER and PR were <1%), HER2 status, disease stage (based on the 8th American Joint Committee on Cancer), and treatments performed (including type of breast surgery and axillary management, radiotherapy, chemotherapy, endocrine therapy, and ovarian suppression). We also collected the type and quantity of care provided (outpatient visits, hospitalizations, drugs, among others), alongside unit and total costs. These data were retrieved from the cancer registry database and electronic medical records (EMRs). Unit costs were obtained from official government price lists and the centre costing system. The patients were followed until 30 June 2023.

### End points

The primary endpoint was to identify the main clinicopathological differences between HER2-0, “HER2-low”, and HER2-positive eBC. Secondary endpoints included overall survival (OS), defined as the time from the date of surgery to the date of death from any cause, and disease-free survival (DFS), defined as the time from the date of surgery to the date of loco-regional or distant recurrence or death from any cause, whichever occurred first. We also assessed direct medical costs per patient from eBC diagnosis to the end of follow-up. For the present analysis, costs were divided into two main categories: treatment-related costs (surgery, radiotherapy, and systemic therapies) and other costs (hospitalization, consultations, blood work, and diagnostic imaging such as CT scans and ultrasounds).

### Statistical analysis

Patient’s characteristics are presented as counts and proportions and compared using the chi-square test, for categorical variables, or as mean and standard deviations and compared using analysis of variance (ANOVA), for continuous variables. DFS and OS were estimated using the Kaplan-Meier method and compared using the log-rank test. All analyses were performed using R version 4.1.2 (R Core Team, Vienna, Austria). Results were considered statistically significant for p-values less than 0.05.

### Ethics

The present study was approved by the Ethics Committee of the IPO-Porto (ref. CES. 009/024). The data comprised no unique personal identifiers and were extracted from the IPO-Porto Cancer Registry database and EMRs. Thus, patient informed consent was not required.

## Results

### Clinico-pathological characteristics

Among the 1,138 patients included in the analysis, 35.1% (n=400) were classified as having HER2–0 eBC, 45.3% (n=516) had “HER2-low” eBC, and 19.5% (n=222) had HER2-positive eBC.

Compassing all HER2-negative eBC (n=916, 80,5%), “HER2-low” represented 56.3% of the patients. Patient and tumor characteristics stratified by HER2 status are detailed in **Table 1** and comparison between two groups are available at **Supplementary Tables 1** and **2**.

**Table 1:** Demographic and clinical characteristics of the sample, overall and according to HER2 group.

Patient and disease characteristics	HER2-0 (n=400)	HER2-low (n=516)	HER2-positive (n=222)	P-value
Age (years)				
Mean (SD)	58 (± 13)	58 (± 13)	55 (± 13)	0.006
Menopausal status, N (%)				
Postmenopause	254 (64)	324 (63)	127 (57)	0.262
Premenopause	146 (36)	192 (37)	95 (43)	
Stage, N (%)				
I	296 (74)	354 (69)	113 (51)	<0.001
II	90 (22)	138 (27)	87 (39)	
III	14 (4)	23 (4)	22 (10)	
Missing	0 (0)	1 (0.2)	0 (0)	
HR status, N (%)				
Negative	43 (11)	32 (6)	65 (29)	<0.001
Positive	357 (89)	484 (94)	157 (71)	
Histologic type, N (%)				
Lobular	59 (15)	59 (11)	8 (4)	<0.001
No special type	301 (75)	426 (83)	206 (93)	
Others	40 (10)	31 (6)	8 (4)	
Histologic grade, N (%)				
1	45 (11)	37 (7)	0 (0)	<0.001
2	208 (52)	248 (48)	58 (26)	
3	145 (36)	230 (44)	163 (74)	
Missing	2 (0)	1 (0)	1 (0)	
Laterality, N (%)				
Bilateral	2 (0)	2 (0)	0 (0)	0.476
Left-sided	194 (48)	277 (54)	117 (53)	
Right-sided	204 (51)	237 (46)	105 (47)	
Nodal disease, N (%)				
No	282 (70)	329 (64)	165 (74)	0.011
Yes	113 (28)	179 (35)	55 (25)	
Missing	5 (1.3)	8 (1.6)	2 (0.9)	

N, number of patients; SD, Standard deviation.

Patients with “HER2-low” eBC had a mean age of 58 years (± 13), similar to HER2–0 patients, but significantly older than those with HER2-positive disease (55 ± 13, p=0.006). Menopausal status did not differ significantly between groups, with more than half of all patients being postmenopausal (*p*=0.262).

Stage distribution differed significantly. “HER2-low” tumors were more frequently diagnosed at an early stage compared to HER2-positive tumors, with 69% vs. 51% of cases at stage I (*p* < 0.001). Compared to HER2–0 disease (74% stage I), “HER2-low” tumors were slightly more advanced, though this difference was not statistically significant (*p*=0.216).

Endocrine receptor (ER and/or PR) positivity was highest in the “HER2-low” group, being significantly greater than in HER2-0 (94% vs 89%, *p*=0.018) and HER2-positive tumors (94% vs 71%, *p*<0.001).

Histologically, HER2-low tumors were predominantly of ductal/no special type, significantly more than HER2-0 (83% vs. 75%, *p*=0.018), but less than HER2-positive disease (83% vs. 93%, p < 0.001). Lobular carcinomas were most prevalent in HER2-0 (15%), followed by HER2-low (11%), and were rare in HER2-positive tumors (4%). Regarding histologic grade, HER2-low tumors showed an intermediate distribution: 7% grade 1, 48% grade 2, and 44% grade 3. Compared to HER2-0, “HER2-low” tumors had a lower frequency of grade 1 disease (7% vs. 11%), and a higher proportion of grade 3 (44% vs. 36%), being statistically different (*p*=0.025). Compared with HER2-positive tumors, which had 74% grade 3, the difference was highly significant (*p*<0.001).

Laterality did not differ across groups (*p*=0.476), with right-sided tumors slightly predominating. Bilateral cases were rare (n=2 overall).

Nodal involvement was more frequent in HER2-low than in HER2–0 patients (35% vs. 28%, *p*=0.041), and also than HER2-positive disease (35% vs. 25%, *p*=0.009).

### Treatment-related characteristics

**Table 2** describes the treatment characteristics according to HER2 group. Comparison with “HER2-low” can be found in **Supplementary Tables 3** and **4**.

**Table 2:** Treatment characteristics of the sample, according to HER2 group.

Type of treatment	HER2-0 (n=400)	HER2-low (n=516)	HER2-positive (n=222)	P-value
Surgery, N (%)				
No	1 (0)	2 (0)	2 (1)	0.487
Yes	399 (100)	514 (100)	220 (99)	
Lymph node surgery, N (%)				
ALND	32 (8)	71 (14)	45 (20)	0.222
SLNB	354 (88)	433 (84)	157 (71)	
TAD	13 (3)	9 (2)	18 (8)	
Missing	1 (0.3)	3 (0.6)	2 (0.9)	
Radiotherapy, N (%)				
No	103 (26)	149 (29)	51 (23)	<0.001
Yes	297 (74)	367 (71)	171 (77)	
Chemotherapy, N (%)				
No	196 (49)	229 (44)	38 (17)	<0.001
Yes	204 (51)	287 (56)	184 (83)	
Anthracycline-based chemotherapy, N (%)				
No	243 (61)	308 (60)	63 (28)	<0.001
Yes	157 (39)	208 (40)	159 (72)	
Endocrine therapy, N (%)				
No	45 (11)	29 (6)	60 (27)	<0.001
Yes	355 (89)	487 (94)	162 (73)	

ALND, axillary lymph node dissection; N, Number of patients; SLNB, sentinel node biopsy; TAD, targeted axillary dissection

Relating to surgery performance and type of lymph node surgery, there were no differences between “HER2-low” and HER2–0 and HER2-positive disease.

However, there were differences in systemic treatment. The use of endocrine therapy was highest in the “HER2-low” group (94% vs. 89% HER2-0, *p*=0.003; and 94% vs 73% HER2-positive, *p*<0.001). Chemotherapy use also differed significantly between “HER2-low” and HER2-positive patients, being higher in the latter (56% vs. 83%, *p*<0.001) but no differences were found between “HER2-low” and HER2–0 in this matter. Anthracycline-based regimens were also more frequent in HER2-positive patients, compared with “HER2-low” (40% vs.72%, *p*<0.001).

### Oncologic outcomes

**Figure 1:**
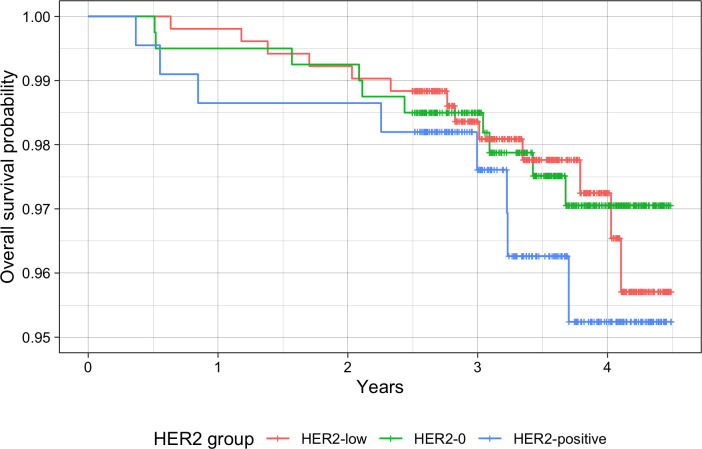
Overall survival in patients with HER2-0 (green), HER2-low (red) and HER2-positive (blue) eBC.

**Figure 2:**
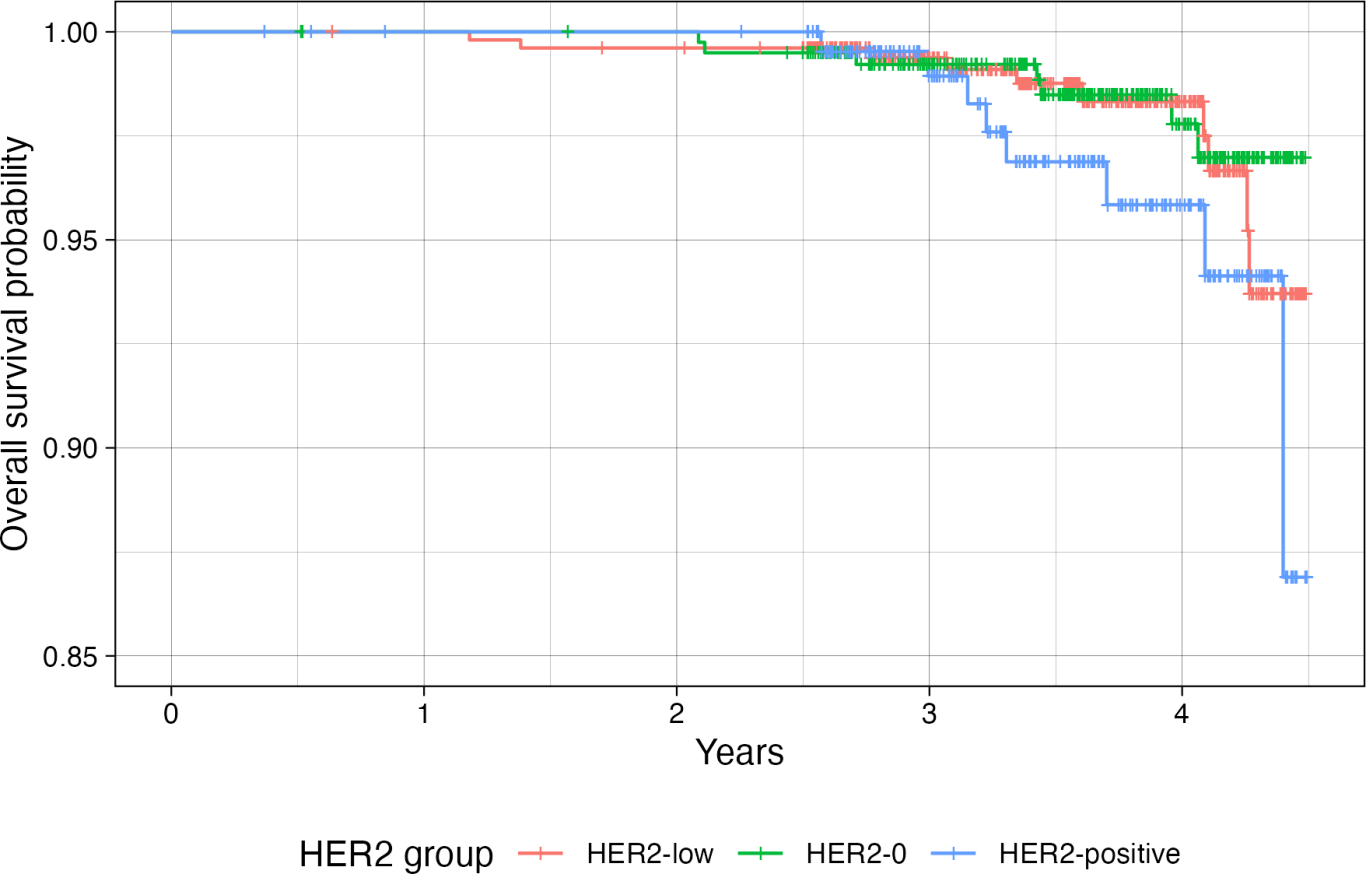
Disease-free survival in patients with HER2-0 (green), HER2-low (red) and HER2-positive (blue) eBC.

The median follow-up time for the population was 43 months (range: 4-54). At the end of follow-up, 31 deaths and 25 disease recurrences were recorded. No significant differences were observed in DFS and OS between “HER2-low” and HER2–0 or HER2-positive disease (**Figures 1**, **2**). A subgroup analysis was not performed due to the limited number of events at this early follow-up.

### Unit costs

**Figure 3:**
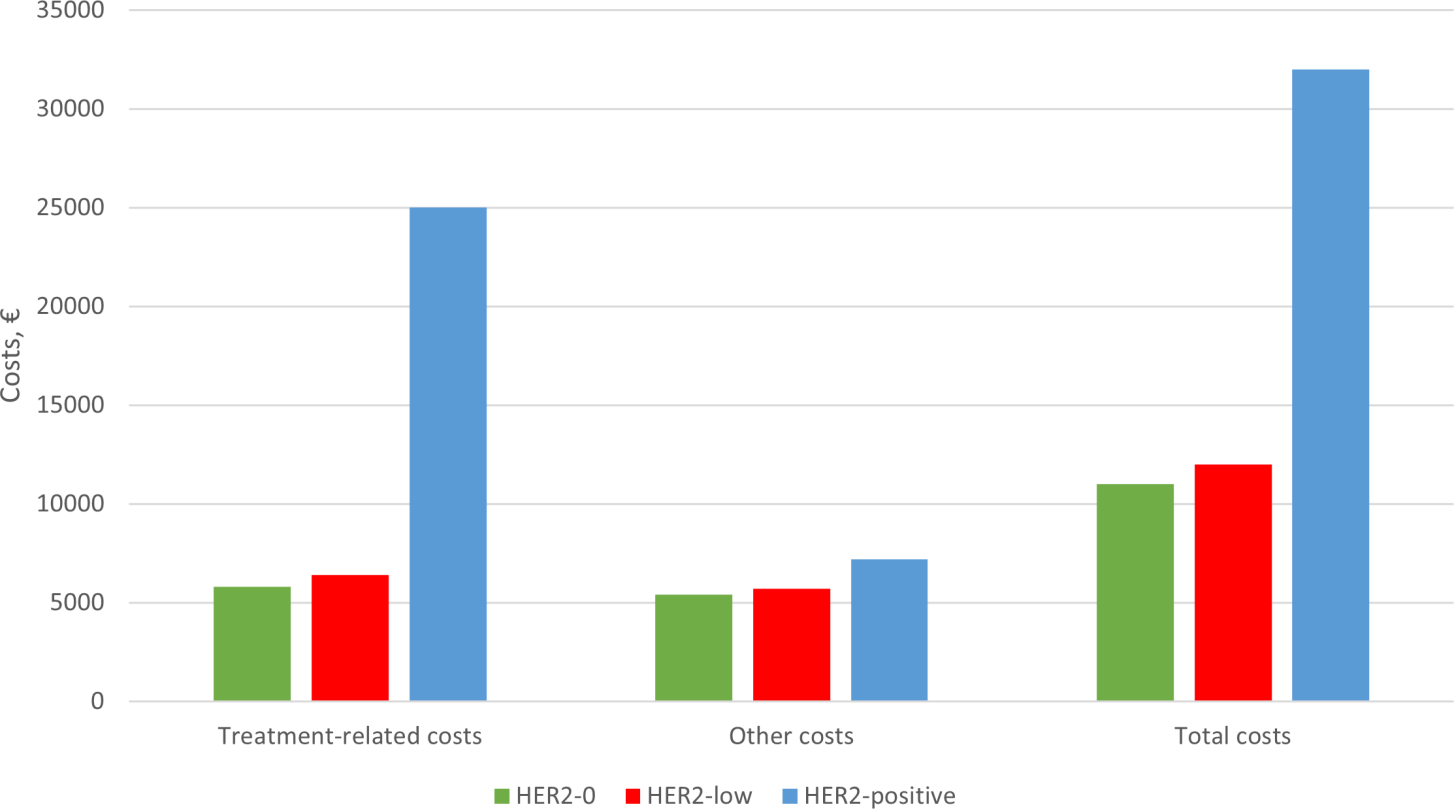
Average treatment costs according to HER2 group (values in euros - €).

The average treatment costs are presented in **Table 3**, with a graphical representation in **Figure 3**.

**Table 3:** Average treatment costs according to HER2 group (values in euros - €).

Type of cost	HER2-0 (n=400)	HER2-low (n=516)	HER2-positive (n=222)	P-value
Medical costs				
Mean (SD)	5200 (± 3000)	5300 (± 3800)	25000 (± 17000)	<0.001
Other costs				
Mean (SD)	6500 (± 3200)	6900 (± 3500)	8900 (± 5900)	<0.001
Total costs				
Mean (SD)	12000 (± 5000)	12000 (± 5800)	34000 (± 19000)	<0.001

N, number of patients; SD, Standard deviation.

In our cohort, HER2-positive disease incurred the highest total costs, with a mean cost of €34,000 per patient, including €25,000 in treatment-related expenses (p<0.001). There were no significant cost differences between the “HER2-low” and HER2–0 groups.

Average treatment costs by stage of disease are presented in **Table 4**. Patients with more advanced clinical stages tended to have higher total costs. Specifically, in stage III disease, the mean costs were €7,700 (±€3,000) for HER2-0, €8,600 (±€7,200) for “HER2-low”, and €36,000 (±€16,000) for HER2-positive disease.

**Table 4:** Average treatment costs according to disease stage (values in euros - €).

Stage of disease	HER2-0 (n=400)	HER2-low (n=516)	HER2-positive (n=222)
Stage I			
Mean (SD)	4600 (± 2800)	4700 (± 3000)	20000 (± 16000)
Stage II			
Mean (SD)	6900 (± 2900)	6500 (± 4400)	28000 (± 18000)
Stage III			
Mean (SD)	7700 (± 3000)	8600 (± 7200)	36000 (± 16000)

N, number of patients; SD, Standard deviation.

## Discussion

Our study aimed to investigate the clinicopathological characteristics, oncologic outcomes, and costs associated with HER2 status in patients with eBC. We found the prevalence of “HER2-low” disease to be 45.3%, consistent with the range reported in other retrospective studies (47.5–56.5%) (7, 9). Among HER2-negative eBC, “HER2-low” accounted for 56.3% of patients.

Differences in age were observed only in the HER2-positive group, which is expected since younger women are more likely to have HER2-enriched tumors (10). However, menopausal status did not differ significantly between the groups.

“HER2-low” disease showed the highest rate of HR positivity among the three groups, which was statistically significant. The predominance of HR in HER2-low eBC has also been reported in other studies, when compared with HER2-0 (5, 7, 11). A study evaluating the expression of PAM50 genes (8) showed a predominance of luminal-related genes in “HER2-low” compared with HER2–0 BC, with no gene profile differences within triple-negative BC. This correlates with the higher prevalence of patients undergoing endocrine therapy, as indicated by our results. Differences between HER2-positive and “HER2-low” tumors are less clearly established.

“HER2-low” eBC was also associated with higher histological grade compared with HER2–0 disease, yet a lower histological grade compared with HER2-positive disease. This aligns with some studies. Di Cosimo et al. (5) analyzed 699 BC patients undergoing neoadjuvant therapy and noted that “HER2-low” BC, compared with HER2-0, tended to have worse histological grade. Park et al. (9) also demonstrated that “HER2-low” had fewer grade 1 tumors than HER2–0 BC. However, Tarantino P et al. (11) showed “HER2-low” had fewer high-grade tumors compared with HER2-0. In our study, HER2 expression seems to follow a gradient, as reflected by the proportion of grade 3 disease: 36% for HER2-0, 44% for “HER2-low”, and 74% for HER2-positive.

“HER2-low” was more often of NST or ductal subtype than HER2–0 eBC, consistent with other studies (11–13). Also, HER2-positive eBC showed an even higher frequency of ductal subtype compared with “HER2-low” eBC. This is consistent with the known lower prevalence of HER2 expression or ERBB2 amplification in invasive lobular carcinomas (14, 15) and the fact that lobular carcinomas more frequently present a HER2–0 phenotype (15).

In our study, no differences in OS or DFS emerged among the three groups. Other reports have also shown no differences between “HER2-low” and HER2–0 in this regard (16, 17), but the literature is still inconsistent. Some investigations indicate a worse prognosis for “HER2-low” eBC (5, 18–20), whereas others report a better 10-year OS compared with HER2-0 (13) or a better prognosis in node-negative disease (21). Therefore, there is no consensus that “HER2-low” is an independent prognostic factor, underscoring the need for more studies. The absence of differences between HER2-negative and HER2-positive disease in our study is less straightforward but could be attributed to the efficacy of anti-HER2 treatments. Moreover, the relatively short follow-up period may not permit definitive conclusions about OS and DFS. Currently, tumors with low to moderate expression but lacking ERBB2 amplification are not considered targetable with conventional anti-HER2 drugs such as trastuzumab, as demonstrated by the NSABP B-47 (22) phase III trial, which found no benefit for trastuzumab in “HER2-low” tumors regarding DFS, distant recurrence-free interval, or OS.

Patients with HER2-positive eBC were more frequently treated with chemotherapy (83% vs 51% for HER2–0 and 56% for “HER2-low”, p<0.001). This aligns with current treatment guidelines, which generally recommend neoadjuvant chemotherapy in combination with trastuzumab and pertuzumab for patients with cT2 or cN+ eBC, followed by surgery and adjuvant trastuzumab with or without pertuzumab or trastuzumab-emtansine (T-DM1), depending on treatment response (23–25). Patients with cT1 are as well candidates to adjuvant treatment with anti-HER2 therapies and chemotherapy (23, 26, 27). Consequently, HER2-positive patients typically undergo systemic therapy. This is likely the main driver of the increased treatment-related costs in patients with HER2-positive eBC compared with “HER2-low” and HER2–0 eBC. Thus far, there are no significant differences in treatment costs between “HER2-low” eBC and HER2–0 eBC.

A key limitation of our study is the short follow-up time for early-stage disease, which limits the number of observed events and impacts the analysis of oncologic outcomes. More time is required to draw firm conclusions about OS and DFS. Moreover, our data came from a single comprehensive oncology center in Northern Portugal, which may limit the generalizability of our findings. Nevertheless, this center is the largest in the country, treating patients from all regions and diverse sociodemographic backgrounds, and its clinical practice follows current guidelines. Therefore, these results likely reflect a broader real-world context despite this constraint.

## Conclusion

In this single-center retrospective analysis, we observed that “HER2-low” eBC displays unique clinicopathological characteristics, notably higher HR positivity compared with HER2–0 eBC and a lower histological grade than HER2-positive disease. Nonetheless, these distinctions did not translate into worse outcomes in terms of DFS and OS, possibly due to the relatively short follow-up and the efficacy of existing adjuvant therapies. Meanwhile, the highest treatment costs were noted among patients with HER2-positive tumors, driven by the expense of targeted agents.

Collectively, our results suggest that “HER2-low” has distinct biological and clinical features, though its prognostic value remains uncertain. Longer follow-up, biomarker-driven research and multicentric studies are needed to better elucidate the role of HER2 status in treatment planning and to determine whether novel targeted strategies could further improve outcomes in this population.

## Data Availability

The datasets presented in this study are not publicly available due to ethical restrictions. Access to the data is limited to the researchers involved in the study as per the ethics committee approval. Requests to access the datasets should be directed to the corresponding author, who will consider them in accordance with the ethical guidelines.
