# Supplementary material for: The clinicopathological characteristics, oncologic outcomes and costs of “HER2-low” early breast cancer compared to HER2-zero and HER2-positive: a single-centre retrospective analysis
Source: Front Oncol. 2025 Sep 17;15:1579602. doi: 10.3389/fonc.2025.1579602 (PMC12483866; doi:10.3389/fonc.2025.1579602)
Supplement: Supplementary file 1 [file Table1.docx]

**Supplemental Table 1. Comparison between HER2-0 and “HER2-low” eBC of demographic and clinical characteristics of the sample**

|  | HER2-0  (n=400) | HER2-low  (n = 516) | p-value |
| --- | --- | --- | --- |
| Age (years) |  |  |  |
| Mean (SD) | 58 (± 13) | 58 (± 13) | 0.888 |
| Menopausal status, N (%) |  |  |  |
| Postmenopause | 254 (64) | 324 (63) | 0.879 |
| Premenopause | 146 (36) | 192 (37) |  |
| Stage, N (%) |  |  |  |
| I | 296 (74) | 354 (69) | 0.216 |
| II | 90 (22) | 138 (27) |  |
| III | 14 (4) | 23 (4) |  |
| Missing | 0 (0) | 1 (0.2) |  |
| HR status, N (%) |  |  |  |
| Negative | 43 (11) | 32 (6) | 0.018 |
| Positive | 357 (89) | 484 (94) |  |
| Histologic type, N (%) |  |  |  |
| Lobular | 59 (15) | 59 (11) | 0.018 |
| No special type | 301 (75) | 426 (83) |  |
| Others | 40 (10) | 31 (6) |  |
| Histologic grade, N (%) |  |  |  |
| 1 | 45 (11) | 37 (7) | 0.025 |
| 2 | 208 (52) | 248 (48) |  |
| 3 | 145 (36) | 230 (44) |  |
| Missing | 2 (0) | 1 (0) |  |
| Laterality, N (%) |  |  |  |
| Bilateral | 2 (0) | 2 (0) | 0.294 |
| Left-sided | 194 (48) | 277 (54) |  |
| Right-sided | 204 (51) | 237 (46) |  |
| Nodal disease, N (%) |  |  |  |
| No | 282 (70) | 329 (64) | 0.041 |
| Yes | 113 (28) | 179 (35) |  |
| Missing | 5 (1.3) | 8 (1.6) |  |

N, number of patients; SD, Standard deviation.

**Supplemental Table 2. Comparison between “HER2-low” and HER2-positive eBC of demographic and clinical characteristics of the sample**

|  | HER2-low  (n = 516) | HER2-positive  (n = 222) | p-value |
| --- | --- | --- | --- |
| Age (years) |  |  |  |
| Mean (SD) | 58 (± 13) | 55 (± 13) | 0.003 |
| Menopausal status, N (%) |  |  |  |
| Postmenopause | 324 (63) | 127 (57) | 0.179 |
| Premenopause | 192 (37) | 95 (43) |  |
| Stage, N (%) |  |  |  |
| I | 354 (69) | 113 (51) | <0.001 |
| II | 138 (27) | 87 (39) |  |
| III | 23 (4) | 22 (10) |  |
| Missing | 1 (0.2) | 0 (0) |  |
| HR status, N (%) |  |  |  |
| Negative | 32 (6) | 65 (29) | <0.001 |
| Positive | 484 (94) | 157 (71) |  |
| Histologic type, N (%) |  |  |  |
| Lobular | 59 (11) | 8 (4) | <0.001 |
| No special type | 426 (83) | 206 (93) |  |
| Others | 31 (6) | 8 (4) |  |
| Histologic grade, N (%) |  |  |  |
| 1 | 37 (7) | 0 (0) | <0.001 |
| 2 | 248 (48) | 58 (26) |  |
| 3 | 230 (44) | 163 (74) |  |
| Missing | 1 (0) | 1 (0) |  |
| Laterality, N (%) |  |  |  |
| Bilateral | 2 (0) | 0 (0) | 0.622 |
| Left-sided | 277 (54) | 117 (53) |  |
| Right-sided | 237 (46) | 105 (47) |  |
| Nodal disease, N (%) |  |  |  |
| No | 329 (64) | 165 (74) | 0.009 |
| Yes | 179 (35) | 55 (25) |  |
| Missing | 8 (1.6) | 2 (0.9) |  |

N, number of patients; SD, Standard deviation.

**Supplemental Table 3.** **Comparison between “HER2-low” and HER2-0 eBC of treatment characteristics of the sample**

|  | HER2-0  (n=400) | HER2-low  (n = 516) | p-value |
| --- | --- | --- | --- |
| Surgery, N (%) |  |  |  |
| No | 1 (0) | 2 (0) | 1 |
| Yes | 399 (100) | 514 (100) |  |
|  |  |  |  |
| Lymph node surgery, N (%) |  |  |  |
| ALND | 32 (8) | 71 (14) | 0.009 |
| SLNB | 354 (88) | 433 (84) |  |
| TAD | 13 (3) | 9 (2) |  |
| Missing | 1 (0.3) | 3 (0.6) |  |
| Radiotherapy, N (%) |  |  |  |
| No | 103 (26) | 149 (29) | 0.329 |
| Yes | 297 (74) | 367 (71) |  |
| Chemotherapy, N (%) |  |  |  |
| No | 196 (49) | 229 (44) | 0.186 |
| Yes | 204 (51) | 287 (56) |  |
| Anthracycline-based chemotherapy, N (%) |  |  |  |
| No | 243 (61) | 308 (60) | 0.797 |
| Yes | 157 (39) | 208 (40) |  |
| Endocrine therapy, N (%) |  |  |  |
| No | 45 (11) | 29 (6) | 0.003 |
| Yes | 355 (89) | 487 (94) |  |

ALND – axillary lymph node dissection; N – Number of patients; SLNB – sentinel node biopsy; TAD - targeted axillary dissection

**Supplemental Table 4.** **Comparison between “HER2-low” and HER2-positive eBC of treatment characteristics of the sample**

|  | HER2-low  (n = 516) | HER2-positive  (n = 222) | p-value |
| --- | --- | --- | --- |
| Surgery, N (%) |  |  |  |
| No | 2 (0) | 2 (1) | 0.746 |
| Yes | 514 (100) | 220 (99) |  |
|  |  |  |  |
| Lymph node surgery, N (%) |  |  |  |
| ALND | 71 (14) | 45 (20) | <0.001 |
| SLNB | 433 (84) | 157 (71) |  |
| TAD | 9 (2) | 18 (8) |  |
| Missing | 3 (0.6) | 2 (0.9) |  |
| Radiotherapy, N (%) |  |  |  |
| No | 149 (29) | 51 (23) | 0.118 |
| Yes | 367 (71) | 171 (77) |  |
| Chemotherapy, N (%) |  |  |  |
| No | 229 (44) | 38 (17) | <0.001 |
| Yes | 287 (56) | 184 (83) |  |
| Anthracycline-based chemotherapy, N (%) |  |  |  |
| No | 308 (60) | 63 (28) | <0.001 |
| Yes | 208 (40) | 159 (72) |  |
| Endocrine therapy, N (%) |  |  |  |
| No | 29 (6) | 60 (27) | <0.001 |
| Yes | 487 (94) | 162 (73) |  |

ALND – axillary lymph node dissection; N – Number of patients; SLNB – sentinel node biopsy; TAD - targeted axillary dissection
